# Making Sense of Patient-Derived iPSCs, Transdifferentiated Neurons, Olfactory Neuronal Cells, and Cerebral Organoids as Models for Psychiatric Disorders

**DOI:** 10.1093/ijnp/pyab037

**Published:** 2021-07-03

**Authors:** Jakob Unterholzner, Vincent Millischer, Christoph Wotawa, Akira Sawa, Rupert Lanzenberger

**Affiliations:** 1 Department of Psychiatry and Psychotherapy, Medical University of Vienna, Austria; 2 Neurogenetics Unit, Department of Molecular Medicine and Surgery, Karolinska Institutet, Stockholm, Sweden; 3 Center for Molecular Medicine, Karolinska University Hospital, Stockholm, Sweden; 4 Department of Mental Health, Johns Hopkins University Bloomberg School of Public Health, Baltimore, MD, USA; 5 Departments of Psychiatry, Neuroscience, Biomedical Engineering and Genetic Medicine, Johns Hopkins University School of Medicine, Baltimore, MD, USA

**Keywords:** Cerebral organoid, iPSC, olfactory neurons, psychiatry, transdifferentiation

## Abstract

The improvement of experimental models for disorders requires a constant approximation towards the dysregulated tissue. In psychiatry, where an impairment of neuronal structure and function is assumed to play a major role in disease mechanisms and symptom development, this approximation is an ongoing process implicating various fields. These include genetic, animal, and post-mortem studies. To test hypotheses generated through these studies, in vitro models using non-neuronal cells such as fibroblasts and lymphocytes have been developed. For brain network disorders, cells with neuronal signatures would, however, represent a more adequate tissue. Considering the limited accessibility of brain tissue, research has thus turned towards neurons generated from induced pluripotent stem cells as well as directly induced neurons, cerebral organoids, and olfactory neuroepithelium. Regarding the increasing importance and amount of research using these neuronal cells, this review aims to provide an overview of all these models to make sense of the current literature. The development of each model system and its use as a model for the various psychiatric disorder categories will be laid out. Also, advantages and limitations of each model will be discussed, including a reflection on implications and future perspectives.

## Introduction

**Figure 1.**
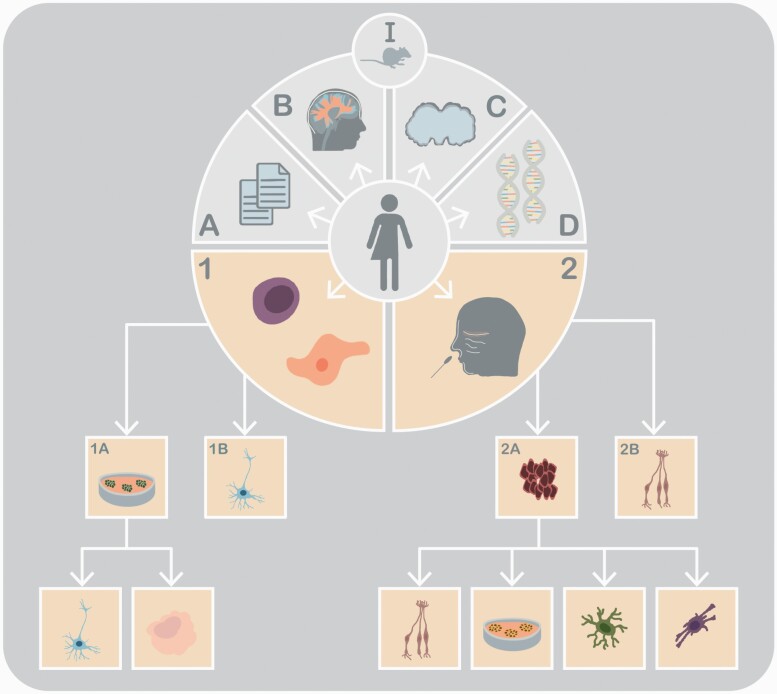
Methods to investigate psychiatric disorders. I: Animal models; (A) psychometric scores, (B) neuroimaging, (C) post-mortem tissue, (D) genetics; (1) somatic cells (including PBMCs, lymphoblastoid cell lines, and fibroblasts) used to generate induced neurons through transdifferentiation (1B) or induced pluripotent stem cells (1A). The iPSCs can then be used to generate different neuronal subtypes types or organoids. (2) Acquisition of olfactory neuroepithelium to obtain olfactory neurons (2B) and olfactory stem cells (2A). These can be used to create neurospheres and different subtypes, including neurons, astrocytes, and oligodendrocytes.

The explanatory model of a disorder is often only as good as the experimental model it is based on. In general, the refinement of experimental models happens through a continuous approximation towards both the location and metabolic signatures of the hypothesized dysregulated cells and tissue. For example, the introduction of vaginal smears or liver biopsies has greatly improved insights into disease mechanisms and the diagnostic and treatment algorithm in the respective fields (Papanicolaou and Traut, 1941; Sherlock, 1945). In psychiatry, an impairment of neuronal structure and function is assumed to play a major role in disease mechanisms and symptom development. Here, the approximation is an ongoing process implicating various fields (illustrated in Figure 1). Animal models enable the investigation of novel neurobiological hypotheses but rarely reflect the syndromal character of most psychiatric disorders (Wang et al., 2017b). Post-mortem cerebral tissue can provide topologically specific biochemical signatures of brain disorders. Autopsied brains are, however, relatively scarce, and confounding psychiatric medication in deceased subjects is common (Story Jovanova et al., 2018). Also, the post-mortem interval is an important factor, potentially affecting the stability and also representability of the tissue (Wang et al., 2017b). Blood has been used predominantly to deduce vulnerability and molecular pathology. It is now possible to perform pharmacogenomic testing for genetic polymorphisms affecting the metabolism of medication using blood samples (Rybakowski and Serretti, 2016). Extrapolating information on brain pathology through blood cells has proven somewhat more difficult, especially since the transcriptome and epigenome of the 2 tissues seem to diverge more than previously presumed (Mele et al., 2015; Walton et al., 2016).

The wish to test hypotheses generated through animal, post-mortem, and genetic studies and to model bio- and neurochemical, cellular, and genetic aspects of psychiatric disorders has led to the development of in vitro models using lymphoblasts and fibroblasts alongside these investigations. Lymphocytes are obtained easily, have a capacity of self-renewal (especially when immortalized using an Epstein-Barr virus modification), and can be used to perform genetic and pharmacogenetic analyses (Welsh et al., 2009). Models generated from lymphocytes continue to be used for investigations of treatment effects (Chierrito et al., 2019), proteomic analyses (Yoshimi et al., 2019), and cell signaling pathways (Viswanath et al., 2015). However, many studies using lymphoblasts as models for psychiatric disorders still await replication. Human-derived dermal fibroblasts are also easily obtainable through skin biopsy, consisting of proliferating and postmitotic cells that can be cultured for multiple passages without loss of genetic stability (Kalman et al., 2016) enabling reproducible experiments. These include studies on signaling pathways and the role of stress as well as age- and pharmacology-dependent mechanisms. Fibroblasts, and also lymphoblasts, have been compared repeatedly with CNS tissue to evaluate compatibility of different peripheral biomarkers for psychiatric disorders (Hayashi-Takagi et al., 2014), with limited success so far. Moreover, no significant gene expression differences were reported in lymphoblastoids and fibroblasts of patients suffering from schizophrenia and healthy controls. In general, these models might rather be of heuristic value and serve as validation methods for genes implicated through techniques using neuronal tissue (Matigian et al., 2008). Consequently, alongside the obvious advantages and the informative value of lymphocyte and fibroblast models, certain limitations must be considered. The immortalization procedure of lymphocytes might result in erasure of epigenetic marks and introduce genetic instability, thereby altering gene expression in relation to the unmodified cells. Regarding fibroblasts, the varying cell stages, the cellular age, and environmental influences need to be taken into account. The differences of specific features between these cells and neuronal cells, however, is still discussed as the most important limitation of these models (Kalman et al., 2016).

The focus thus currently lies on the “right” in vitro experimental model to study psychiatric disorders. Being considered brain circuit disorders (Insel and Cuthbert, 2015), the logical step is using cells with neuronal signatures, especially since large consortium studies repeatedly implicate genes involved in neuronal development and synaptic function in the pathophysiology of schizophrenia (SCZ), bipolar disorder, major depressive disorder (MDD), anorexia nervosa, autism spectrum disorder (ASD), and Gilles de la Tourette syndrome (Lee et al., 2019). Neuronal cell models are thus indispensable not only to replicate these results but also to generate new hypotheses that might contribute to more specific explanatory models for psychiatric disorders. Building upon insights gained from research using embryonic stem cell and modified human metastatic neuroblastoma tissue cell lines and using a set of transcription factors, induced pluripotent stem cells (iPSCs) can now be generated from human somatic cells. These can then be cultured to form neuronal precursor cells and subtypes. Moreover, iPSCs can be cultured to form 3-dimensional cerebral organoids. Also, various somatic cells can be induced directly to form specific neuronal subtypes via a process called transdifferentiation. Another source of neuronal tissue comprises the olfactory mucosa, where both adult neurons and olfactory neuronal stem cells can be found. Combined, iPSCs, transdifferentiated neurons (iNs), olfactory neurons, olfactory stem cells, and cerebral organoids might represent an additional part to complement the current methods and to further the discovery of new disease models and improve the current diagnostic and therapeutic algorithm (illustrated in Figure 1).

While the clinical nosology of psychiatric disorders continues to be refined, various aspects remain highly relevant when trying to develop explanatory models, including age of onset (neurodevelopmental vs neurodegenerative), different disease trajectories (episodic, chronic, remitted, residual), the assumed underlying neurotransmitter systems (e.g., dopaminergic, serotonergic, noradrenergic, glutamatergic) and brain regions (prefrontal cortex, hippocampus) as well as potential genetic and environmental effects. The most viable model for each disorder would need to incorporate these specific aspects and might encompass a combination of neuronal cells to study both trait characteristics and state changes. Optimally, the tissue would also have to be easily obtainable and adequate for use in an outpatient setting.

Models using iPSCs and neurons derived from them—iNs, cerebral organoids, and olfactory neuronal cells—are becoming increasingly important, highlighted by the plethora of studies in the last decade. This review aims to provide a starting point to gain insights into all these models and to make sense of the current literature. The development of each model system and its use as a model for the various disorder categories will be laid out. Where necessary and adequate, references will be made to more extensive reviews on specific topics. We will also discuss advantages and limitations of each model and reflect on implications and future perspectives.

## Neuronal Models for Disorders of the Brain

Psychiatric disorders are influenced by the genetic and epigenetic information, molecular, cellular, and neurochemical compositions, systemic pathways, and psychological and social aspects (Kalman et al., 2016) that converge in a dysfunction of brain structure and function. As brain network disorders, the pathology affects the whole brain, but each individual disorder also shows alterations in specific brain areas or neurotransmitter systems. Consequently, depending on the disorder, neurons from different sources have been proposed as experimental models.

### Patient-Derived iPSCs

#### Development of the Model

**—**Based on the work of Takahashi and Yamanaka (Takahashi and Yamanaka, 2006, Takahashi et al., 2007), it is possible to generate iPSCs through the expression of various transcription factors (*Klf4*, *Oct4*, *Sox2*, *c-Myc*) using easily accessible somatic cells such as fibroblasts or hematopoietic cells. By way of defined protocols, these iPSCs can then be differentiated into specific neuronal subtypes (Wen et al., 2016) and cultured to form complex neural architectures (Karus, 2014). Once differentiated, in vitro disease modeling can be performed and thereby also drug development and toxicology screens as well as potential biomarker identification (Soliman, 2017). Combining iPSCs with CRISPR/Cas9 gene editing technology could assess the impact of single nucleotide polymorphisms (SNPs) and non-coding variants on neuronal function and architecture (Wen, 2016).

Fibroblasts currently still represent the main source for the generation of iPSCs (Raab et al., 2014). However, obtaining the cells through skin biopsy is considered a laborious process, with the need for culturing of the cells prior to reprogramming. While using lymphocytes was associated with low reprogramming efficiency, newer techniques were shown to reliably create iPSCs and neurons from lymphocytes and to model neurological diseases (Fujimori et al., 2016; Kumar et al., 2020). Compared with fibroblasts, using lymphocytes for reprogramming requires approximately 0.5 mL of blood and is minimally invasive. Also, lymphocytes represent an almost unlimited source of proliferative material for reprogramming, where large repositories are available (e.g., ASDs), which include genotypic and phenotypic data that could be used for iPSC research with these optimized technologies (Walker et al., 2021).

In general, the process of differentiation involves the formation of embryoid bodies from iPSCs as a first step (Russo et al., 2015). From these embryoid bodies, a neural rosette and consequently neuronal progenitor cells and neuronal subtypes can be generated with specific growth factors in a monolayer culture (Logan et al., 2019). By way of standardized protocols, further cultivation of embryoid bodies can lead to the formation of ventricular zone and subventricular zone-like regions (Karus et al., 2014). Since the differentiation process involves the creation of neuronal stem cells as an intermediate step, different receptor subtypes can also be generated in parallel.

Considering the importance of different neuronal subtypes for specific psychiatric disorders, some protocols will be highlighted in the following paragraphs, including dorsal and ventral forebrain neurosphere cultures and monoaminergic and hippocampal neurons. For a more detailed description of current protocols to generate neuronal and non-neuronal cells, please see (Wen et al., 2016; Logan et al., 2019). Liu et al. (2013) provided an efficient protocol for the generation of forebrain GABA interneurons from iPSCs. This protocol was optimized to generate GABA-ergic interneurons from iPSCs that could form functional synapses with induced glutamatergic neurons and integrate into neuronal networks in vitro (Sun et al., 2016). Different groups have managed to improve both sample purity and time cost for generating GABA-ergic interneurons since then (Barretto et al., 2020; Shen et al., 2020), which could help confirm insights gained from post-mortem studies (Rajkowska et al., 2007). Models for the GABA-ergic system appear especially relevant for the investigation of substance use disorders, anxiety disorders, and panic disorders, but also for the subtype of anxious depression and SCZ.

One promising model for affective disorders, anxiety disorders, and attention deficit and hyperactivity disorder (ADHD) might be iPSCs-derived monoaminergic neurons when considering symptoms like sadness, loss of pleasure, increased anxiety, and attention as neurotransmitter specific (Nutt, 2008) and the mechanism of action of most current antidepressants, anxiety, and ADHD medication. Using iPSCs generated from fibroblasts (as well as human embryonic stem cells), Lu et al. (2016) were among the first to generate central serotonin neurons that express key serotonin markers and that respond to the selective serotonin reuptake inhibitor (SSRI) escitalopram, with a dose-dependent increase of serotonin concentration, implicating their technique as a potential serotonergic drug screening assay. Researchers of the same group went on to demonstrate the utility of these iPSC-derived neurons for the study of the human serotonergic system by showing the reactivity of these neurons to the serotonin-specific toxin 5,7-DHT and their functionality after transplantation into the fourth ventricle of new-born mice (Cao et al., 2017). Using embryonic stem cells, but not iPSCs, Mong et al. (2014) have generated noradrenergic neurons and showed that imipramine, an antidepressive drug acting on noradrenergic receptors, bound monoaminergic receptors of these neurons. Midbrain dopaminergic neurons generated from iPSCs by Kriks et al. were shown to be engraftable in a model of Parkinson’s disease (Kriks et al., 2011). Regarding psychiatric disorders, iPSC-derived dopaminergic neurons have so far been predominantly used for the study of SCZ and bipolar disorders (Sauerzopf et al., 2017), but, through the link of the dopaminergic system to reward, pleasure, and motivation (Nutt, 2008), these neuronal subtypes could also be applied as models for MDD and ADHD. Regarding hippocampal neurons, Yu et al. (2014) were able to model hippocampal neurogenesis using iPSC-derived cells and to show the formation of a functional neuronal network when culturing the cells on hippocampal astrocytes.

#### Patient-Derived iPSCs as Models for Disorders of the Brain

**—**Since the development of iPSC-derived neurons, studies using them have focused on the effect of highly penetrant mutations on neuronal structure and function, on stem-cell based therapies for neurodegenerative disorders, and on complex psychiatric disorders and their disorder-specific cellular phenotypes. The last decade has seen a rapid increase in publications. Regarding SCZ alone, more than 50 studies have been published in the last 10 years. McNeill et al. (2020) performed a systematic review of iPSC models for psychiatric disorders recently. In this section, we therefore highlight the major findings while including relevant studies published since then (see Table 1 for an overview of studies as well as reviews on models using iPSCs).

**Table 1.** Selection of Findings From iPSC-Derived Models for Psychiatric Disorders

Disorder	Model	Results	Reference
Neurodegenerative disorders			
	iPSC-derived neurons of different origins	Insights into mechanisms of release, uptake, and toxicity of disease-associated proteins, including α-Synuclein, tau, amyloid-β, huntingtin, and TDP-43	(de Rus Jacquet et al., 2021) (review)
Substance use disorders			
Alcohol	iPSC-derived neurons of different origins	Altered NMDA-receptor activity and involvement of different subunits of the GABA-A receptor in the pathomechanisms of alcohol use disorder	(McNeill et al., 2020) (review)
	Astrocytes and forebrain neurons generated from fibroblast-derived iPSCs	Downregulation of TSPAN5 and similar effects of ethanol and acamprosate on serotonin concentrations in culture	(Ho et al., 2020)
	Forebrain neurons generated from fibroblast-derived iPSCs	Changes of gene expression associated with cholesterol homeostasis through alcohol	(Jensen et al., 2019)
Cannabis	Cortical neurons generated from keratinocyte-derived iPSCs	iPSC-derived cortical neurons predominantly express the cannabinoid type 1, which responds to exogeneous cannabinoids; THC affects neurite outgrowth	(Shum et al., 2020)
	Forebrain neurons generated from fibroblast-derived iPSCs	Dampened cellular and molecular phenotype through THC; changes in THC-associated genes also implicated in psychiatric disorders	(Guennewig et al., 2018)
Opioids	GABA-ergic neurons generated from primary lymphocyte-derived iPSCs (in combination with CRISPR/Cas9	SNP of µ-opioid receptor (N40D) affects spontaneous inhibitory currents	(Halikere et al., 2020)
Schizophrenia			
	iPSC-derived neurons of different origins	Alterations in synaptic transmission, energy metabolism and disturbed neuronal development	(Sauerzopf et al., 2017) (review)
	Cortical neurons generated from fibroblast-derived iPSCs in combination with CRISPR/Cas9	Mutation of DISC1 affects interaction with ATF4 on structural and molecular levels	(Wang et al., 2021)
	iPSCs derived from PBMCs	Feasibility of generating iPSCs from PBMCs with an exonic deletion of ASTN2, with potential to differentiate into 3 germ layers	(Arioka et al., 2018)
	Cortical neurons generated from PBMC-derived iPSCs	Reelin gene mutation (deletion) associated with increased neuronal cell death	(Arioka et al., 2020)
	iPSCs derived from PBMCs	Feasibility of generating iPSCs from PBMCs with de novo mutations in KHSRP, LRRC7, and KIR2DL1, with potential to differentiate into 3 germ layers	(Hathy et al., 2021)
	Forebrain neurons and oligodendrocyte progenitor cells generated from fibroblast-derived iPSCs	Abnormal cellular morphology and myelination potential in iPSC derived OPCs with 2 missense mutations in the CSPG4 gene of patients with schizophrenia	(de Vrij et al., 2019)
	Cortical neurons generated from fibroblast-derived iPSCs (and induced microglia-like cells derived from PBMCs)	Increased synapse elimination and synaptic pruning through microglia in cortical neurons from patients with schizophrenia, improved through minocycline	(Sellgren et al., 2019)
	Glutamatergic neurons generated from fibroblast-derived iPSCs	Persistent changes in iPSC-derived interneurons through microglia	(Park et al., 2020)
	Glutamatergic neurons generated from fibroblast-derived iPSCs	Aberrant arborization and synaptic density in neurons from patients with schizophrenia, rescued with inhibitor of protein kinase C inhibitor	(Shao et al., 2019)
	NPCs generated from fibroblast-derived iPSCs	Method to perform drug screening using transcriptomic profile changes when applying 135 different drugs	(Readhead et al., 2018)
Affective disorders			
Bipolar disorder	iPSC-derived neurons of different origins	Dysregulations of neurodevelopmental and electrophysiological aspects	(Hoffmann et al., 2018) (review)
	Forebrain neurons generated from fibroblast-derived iPSCs	Effect of lithium on calcium signaling, potentially useful for prediction of treatment response	(Chen et al., 2014)
Major depressive disorder	Hindbrain serotonergic neurons generated from fibroblast-derived iPSCs	Association of both altered growth and morphology of serotonergic neurons and SSRI-resistance in MDD patients	(Vadodaria et al., 2019a)
	Forebrain neurons (mixture of glutamatergic and GABAergic) generated from fibroblast-derived iPSCs	Serotonin-induced postsynaptic neuronal hyperactivity in non-remitters	(Vadodaria et al., 2019b)
Neurodevelopmental disorders			
ASDs	iPSC-derived neurons of different origins	Modelling of neurodevelopment and drug discovery using iPSC-derived neurons in neurodevelopmental disorders	(Wen, 2017) (review)
	iPSC-derived neurons of different origins	Approaches to model ASD using iPSC-derived neurons (and organoids)	(Ilieva et al., 2018) (review)
	iPSC-derived neurons of different origins	Implication of calcium signaling, electrophysiology, cell proliferation, and synaptic density as potential disease phenotypes	(Pintacuda et al., 2021) (review)
	Motorneurons generated from iPSC	Impairment of neuromuscular junction maturation through SHANK3	(Lutz et al., 2020)
	NPCs and glutamatergic and GABA-ergic neurons generated from SHED	Dysregulations of specific modules connected to protein synthesis and synapse/transmission in NPCs and neurons	(Griesi-Oliveira et al., 2020)
ADHD	iPSCs generated from urine epithelial cells	Feasibility of generating iPSCs from urine epithelial cells, that can be used as a model for ADHD in the future	(Sochacki et al., 2016)
	iPSCs generated from fibroblasts	Feasibility of generating iPSCs with a SLC2A3 mutation from fibroblasts, that could be differentiated into all 3 germ layers, as a model for ADHD in the future	(Jansch et al., 2018)
	iPSCs generated from PBMCs	Feasibility of generating iPSCs from PBMCs, that can be used as a model for ADHD in the future	(Tong et al., 2019)
	iPSCs generated from PBMCs and keratinocytes (of children and adolescents aged 6–18)	Feasibility of generating iPSCs from PBMCs and keratinocytes, that can be used as a model for ADHD in the future	(Grossmann et al., 2021)
	NPC generated from keratinocyte-derived iPSCs	Generation of NPCs from keratinocyte-derived iPSCs to study molecular and cellular processes in ADHD	(Re et al., 2018)
	Midbrain dopaminergic neurons generated from fibroblast-derived iPSCs (with a CNV in the PARK2 locus)	Alterations in mitochondrial dynamics through PARK2 CNV, which might impact neuronal development	(Palladino et al., 2020)

Abbreviations: ADHD, attention deficit and hyperactivity disorder; ASD, autism spectrum disorder; ASTN2, Astrotactin 2; ATF4, Activating transcription factor 4; CNV, copy number variation; CRISPR/Cas9, Clustered Regularly Interspaced Short Palindromic Repeats/ CRISPR associated protein 9; CSPG4, Chondroitin Sulfate Proteoglycan 4; DISC1, disrupted in schizophrenia 1; iPSC, induced pluripotent stem cells; NPC, neuronal progenitor cells; GABA, gamma-aminobutyric acid; KHSRP, KH-Type Splicing Regulatory Protein; KIR2DL1, killer cell immunoglobulin like receptor, two Ig domains and long cytoplasmic tail 1; LRRC7, Leucine Rich Repeat Containing 7; MDD, major depressive disorder; NMDA, N-methyl-D-aspartate; OPC, oligodendrocyte progenitor cells; PBMC, peripheral blood mononuclear cells; PARK2, parkin; SHANK3, SH3 And Multiple Ankyrin Repeat Domains 3; SHED, stem cells from human exfoliated deciduous teeth; SLC2A3, Solute Carrier Family 2 Member 3; SNP, single nucleotide polymorphism; SSRI, selective serotonin reuptake inhibitor; TDP43, TAR DNA-binding 43 protein; THC, Δ9-tetrahydrocannabinol; TSPAN5, Tetraspanin 5.

##### Neurodegenerative Disorders

Studies using human iPSCs to model neurodegenerative disorders have focused on the disease-associated proteins, including α-Synuclein, tau, amyloid-β, huntingtin, and TAR DNA-binding 43 protein, and mechanisms regarding their release, uptake, and toxicity, all of which have been reviewed recently (de Rus Jacquet et al., 2021). The biochemical and functional immaturity and the lack of an appropriate microenvironment currently represent the greatest limitations and challenges for studies of neurogenerative disorders using iPSCs. These might be overcome by technologies using 3-dimensional modeling systems (cerebral organoids, see below).

##### Substance Use Disorders

As the most prevalent substance use disorder, studies using iPSC-derived neurons have so far focused on alcohol use disorders (AUD), implicating an altered NMDA receptor activity and different subunits of the GABA_A_ receptor in the pathomechanisms of the disorder (McNeill et al., 2020). In a study using iPSC-derived forebrain-specific neurons and astrocytes from patients suffering from AUD, similar effects of ethanol and acamprosate, an NMDA-antagonist, on serotonin concentrations in culture medium and downregulation of TSPAN5 were described, thereby potentially defining a new mechanism of action of acamprosate (Ho et al., 2020). Also, alcohol was shown to change expression of genes associated with cholesterol homeostasis in a mixed culture of cortical neurons (Jensen et al., 2019). The authors could not detect differences between neural samples of patients and controls, highlighting both the need for adequate sample sizes and potential effects of epigenetic mechanisms (Goetjen et al., 2020).

IPSC-derived neuronal models also represent a good model to study the effects of Δ(9)-tetrahydrocannabinol (THC) on neuronal maturation. Neurons generated from keratinocyte-derived iPSCs seem to express the cannabinoid type 1, but not type 2, receptor, and the type 1 receptor responds to exogeneous cannabinoids (Shum et al., 2020). Exposure to THC has also been shown to result in alterations in neurotransmitter signaling and changes in expression of genes connected to neurodevelopmental disorders (Guennewig et al., 2018).

Regarding opioid use disorders, iPSC-derived inhibitory neurons have been created to assess the impact of an SNP of the µ-opioid receptor (N40D) on synaptic transmission. It could be shown that this polymorphism results in differences of spontaneous inhibitory synaptic currents that might contribute to explain opioid dependence (Halikere et al., 2020).

##### SCZ *Spectrum and Other Primary Psychotic Disorders*

Brennand et al. (2011) were the first to use iPSC-derived neurons as a model for schizophrenia (SCZ). Different genes implicated in SCZ have been studied since then using iPSC-derived neurons, including disrupted-in-schizophrenia 1 (DISC1) (Wilkinson et al., 2019; Wang et al., 2021), astrotactin-2 (ASTN2) (Arioka et al., 2018), and reelin (RELN) (Arioka et al., 2020). In addition, protocols have been set up to combine exome sequencing with iPSC technology to evaluate the effect of de novo mutations on molecular and cellular aspects of neuronal development in a patient suffering from SCZ (Hathy et al., 2021).

Another focus in SCZ research using iPSCs concerns the hypothesis of altered oligodendrocyte-microglia processes (Raabe et al., 2019). Using iPSC-derived oligodendrocyte progenitor cells, abnormal cellular morphology and myelination potential in cells with 2 missense mutations of the chondroitin sulphate proteoglycan 4 were found. Diffusion tensor imaging of the individuals with these mutations showed reduction of white matter integrity compared with unaffected controls (de Vrij et al., 2019). Increased synaptic pruning through microglia as one reason for reduced synapse density was investigated using iPSC-derived cortical neurons and induced microglia-like cells. The authors indeed found increased synapse elimination in cells from patients suffering from SCZ and also implicated minocycline, an antimicrobial chemotherapeutic drug, in the reduction of synapse elimination through microglia (Sellgren et al., 2019). This association was further corroborated by showing persistent changes through microglia in iPSC-derived interneurons of patients suffering from SCZ (Park et al., 2020). Cortical interneurons generated from iPSCs of patients with SCZ also exhibit aberrant arborization and synaptic density, a phenotype that seems to be corrected when applying an inhibitor of protein kinase C (Shao et al., 2019).

Regarding therapeutic agents and discovery, a proof-of-concept approach was proposed using patient-derived neuronal progenitor cells (NPCs) and neurons to assess drug-induced transcriptional signature changes that can be used for drug discovery in the future. The authors also highlighted the advantages of using patient-specific neuronal tissue for drug screening compared with other cells (Readhead et al., 2018).

Also, studies have investigated alterations in synaptic transmission and energy metabolism as well as disturbed neuronal development, reviewed elsewhere (Sauerzopf et al., 2017). While certain limitations still remain (see below), great advances have already been made in increasing sample homogeneity where necessary (Park et al., 2021) and sharing protocols to improve reproducibility and generalizability of results (Stock et al., 2020).

##### Affective Disorders

Due to its high heritability estimates, bipolar affective disorder ranges among the psychiatric disorders most frequently studied using neurons derived from iPSCs. So far, dysregulations of neurodevelopmental and electrophysiological aspects have been described, reviewed by, for example, (Hoffmann et al., 2018). Some of these insights were put into the context of treatment effects. For example, using iPSC-derived forebrain neurons, Chen et al. (2014) could show an effect of lithium on calcium signaling, an insight that might prove useful to predefine lithium responders. Recently, circadian rhythm deficits were implicated in lithium nonresponders in a model using neuronal precursor cells and glutamatergic neurons derived from iPSCs from patients with bipolar disorder (Mishra et al., 2021).

Vadodaria et al. (2019a) were the first to generate iPSC-derived hindbrain serotonergic neurons from a population of patients with MDD divided into SSRI remitters and nonremitters. Their data suggest an association between altered growth and morphology of serotonergic neurons and SSRI resistance in patients with MDD. Also, Vadodaria et al. (2019b) have used iPSC-derived forebrain neurons from a population of patients with MDD (specifically, a mixture of predominantly glutamatergic, but also GABA-ergic neurons) and found a serotonin-induced postsynaptic neuronal hyperactivity in SSRI nonremitters, which was connected to an upregulation of the serotonergic receptors 5-HT2A and 5-HT7. Forebrain-specific neural progenitor cells and predominantly glutamatergic, but GABA-ergic and dopaminergic neuronal subtypes were also created using iPSCs of a patient with a frameshift DISC1 mutation and a diagnosed MDD (Wen et al., 2014) that showed dysregulated protein transcription and synaptic vesicle transport in neurons with the DISC1 mutation compared with control subjects.

In an effort to better characterize functional aspects of a novel gene associated with SSRI treatment response, ERICH3 (glutamate-rich 3), iPSC-derived dopaminergic neurons were used, among other techniques (Liu et al., 2020). The authors found ERICH3 to be colocalized with dopamine in these neuronal subtypes. While that study represents only one further step in understanding the mechanisms of ERICH3, it highlights the potential of iPSC research as a complementary technique to test hypotheses generated through, for example, genome-wide association studies.

##### Neurodevelopmental Disorders

Since iPSCs can be used to recapitulate early stages of cortical development, these cells appear relevant as models for neurodevelopmental disorders, such as ASD and ADHD (Wen, 2017). This is reflected in the plethora of iPSC studies dedicated to this topic, reviewed by, for example, Ilieva et al. (2018). Using iPSC-derived neurons, it is now possible to investigate the effect of highly penetrant mutations on neuronal development in, for example, patients suffering from Rett or Timothy syndrome, 2 forms of ASD. In patients with sporadic forms, iPSCs represent a valuable method to investigate disease mechanisms in vitro. So far, studies have found phenotypes connected to calcium signaling, electrophysiology, cell proliferation, and synaptic density (Pintacuda et al., 2021). Of note, among the many genes studied, various groups have focused on mutations of SHANK3 and SHANK2, structural proteins located at the postsynaptic density (Chen et al., 2020; Lutz et al., 2020). Moreover, results generated from iPSC-derived neurons can be compared with post-mortem data and potentially lead to disease-relevant biomarkers (Griesi-Oliveira et al., 2020). Also, iPSCs and NPCs might be relevant to inform discovery of drugs that specifically target genes implicated in disease mechanisms (Dhindsa et al., 2021).

Regarding ADHD, different cell lines were used to generate iPSCs, including urine epithelial cells of 3 adult patients (Sochacki et al., 2016), fibroblasts of 1 patient carrying a duplication of the neuronal glucose transporter-3 (SLC2A3) (Jansch et al., 2018), and keratinocytes as well as peripheral blood mononuclear cells of several teenage patients (Tong et al., 2019; Grossmann et al., 2021). Re et al. (2018) were able to generate differentiated neurons using hair-derived keratinocytes of 1 adult patient. Palladino et al. (2018) investigated a candidate gene for ADHD, PARK2, coding for a protein involved in mitochondria functioning, using human dermal fibroblasts and iPSC-derived dopaminergic neurons from patients suffering from ADHD. They found that carrying a copy number variation of PARK2 might impact mitochondrial dynamics and thereby processes important during developmental periods (Palladino et al., 2020). Considering their results, they suggest substances affecting mitochondria function (antioxidants) as potential treatment options.

#### Limitations

**—**To facilitate cellular totipotency, the reprogramming procedure to generate iPSCs involves both the resetting of the lineage identity and the erasure of the epigenetic memory of the cell (Soliman et al., 2017). Pronounced environmental influences, that is, disorder-relevant epigenetic marks from external factors such as adverse events or diet, might therefore be lost in the process. The extent of the changes and the comparability with adult neurons will require further study. Directly inducing neuronal subtypes from fibroblasts through transdifferentiation (see below) could reduce the epigenetic erasure and retain the cellular age (Mertens et al., 2018). Reprogramming also includes rejuvenation of the cells. iPSC-derived neurons rather resemble those of the fetal brain, thereby complicating the generation of an adequate model for disorders with onset predominantly in adult life (such as MDD or neurodegenerative diseases). Changing the cellular age (Studer et al., 2015), for example, through the overexpression of progerin (Miller et al., 2013), has therefore become an important goal in iPSC research. Cell heterogeneity is another issue when generating neurons from iPSCs. This heterogeneity concerns both intra- and inter-donor variations of iPSC lines. Fluorescent-activated cell sorting and re-culturing the cells could help in this regard. Also, reprogramming systems that are fully robotic have been introduced to improve variability and the time- and cost-effectiveness of the process (Soliman et al., 2017). To achieve an adequate power might, however, require raising the sample size to above 25 000 subjects (Fromer et al., 2016). When grown in a 2-dimensional monolayer, iPSC-derived neuronal cultures lack the complexities of the human brain and cannot be used to study neuronal circuits (McNeill et al., 2020). Nevertheless, iPSC-derived neuronal 2-dimensional models bear the potential for high-throughput drug testing (Logan et al., 2019) and have additive value in personalized precision psychiatry.

### Transdifferentiation (Functional-Induced Neurons)

#### Development of the Model

**—**Transdifferentiation describes the process of directly generating neurons without the intermediary pluripotent stem cell stage. Using the 3 transcription factors *Ascl1*, *Brn2*, and *Myt1l*, it is possible to induce functional excitatory cortical neurons from fibroblasts (Vierbuchen et al., 2010). Proof-of-principle studies followed describing the successful induction of different neuronal subtypes from fibroblasts, including excitatory, dopaminergic, and motor neurons (Yang et al., 2011). Since these represent terminally differentiated cells, a method to directly reprogram fibroblasts to NPCs (iNPCs) was developed using the transcription factors *Oct4*, *Sox2*, *Klf4*, and *c-Myc* and a transient induction of these factors (Kim et al., 2011). Similar to iPSCs, neural rosette cells and different neuronal subtypes and astrocytes could be generated from these iNPCs. The conversion of fibroblasts to neurospheres can also be achieved using non-neuronal progenitor transcription factors such as *Ptf1a* (Xiao et al., 2018) that exhibit tripotent differentiation potential (i.e., into neurons, astrocytes, and oligodendrocytes) and little tumorigenic risk. INPCs can also be generated from human peripheral blood cells using an *Oct4*-free approach (Sheng et al., 2018). These iNPCs also show tripotency, with age-associated epigenetic marks largely erased. In addition to NPCs, excitatory, motor, and dopaminergic neurons, the conversion of fibroblasts to both noradrenergic (Li et al., 2019) and serotonergic neurons (Vadodaria et al., 2016; Xu et al., 2016) has recently been shown. These serotonergic iNs showed serotonin release in in vitro conditions and responsiveness to SSRIs (Vadodaria et al., 2016). Even though these (especially serotonergic or noradrenergic) neurons would not yet serve a diagnostic purpose, they could be used to predict treatment response, especially since iNs retain aspects, such as age-related markers, of the donor cell’s epigenetic memory (Yang et al., 2011; Mertens et al., 2016). This does not hold true for NPCs generated through transdifferentiation.

#### iNs as Models for Disorders of the Brain

**—**So far, iNs have been predominantly used for age-related neurodegenerative disorders, such as amyotrophic lateral sclerosis or Huntington’s disease, reviewed elsewhere (Traxler et al., 2019).

##### Neurodegenerative Disorders

In patients suffering from Parkinson’s disease, fibroblasts were induced to create functional dopaminergic iNs (Caiazzo et al., 2011), which might also have therapeutic potential (see Table 2). Similar to dopaminergic neurons generated from iPSCs (Gage and Temple, 2013), dopaminergic iNs could be used to study responsiveness to antipsychotic drugs or sensitivity to amphetamines in SCZ and compared with neuroimaging data (Weidenauer et al., 2020) in the future.

**Table 2.** Selection of Findings From Induced Neurons for Psychiatric Disorders

Disorder	Model	Results	Reference
Neurodegenerative disorders			
Parkinson’s disease	iDA	Successful induction of functional dopaminergic neurons from human fibroblasts as a model for Parkinson’s disease	(Caiazzo et al., 2011)
Neurodevelopmental disorders			
ASD	iPSC-iNs	Transdifferentiation prevents manifestation of ASD-associated phenotypes	(Schafer et al., 2019)

Abbreviations: ASD, autism spectrum disorder; iDA, induced dopaminergic neurons; iNs, induced neurons; iPSC, induced pluripotent stem cells.

##### Neurodevelopmental Disorders

iNs (and iPSCs) were used in patients diagnosed with ASD (Schafer et al., 2019). It was shown that a disorder-related phenotype was present only in the process of iPSC differentiation but not in iNs, highlighting the neurodevelopmental aspects of the ASD-specific pathology (see Table 2).

#### Limitations

**—**The generation of transdifferentiated serotonergic neurons still requires expertise and experience, taking up to 6 weeks (Vadodaria et al., 2016). Also, the donor cell and the genetic mosaicism should be considered in this regard (Mertens et al., 2016). Future studies should compare iNs from the same type of donor cell with neurons in different developmental stages.

### Olfactory Neurons and Neuronal Stem Cells

#### Development of the Model

**—**The olfactory epithelium consists of sustenacular and microvillar cells, olfactory neurons, and different types of olfactory stem cells (Graziadei and Graziadei, 1979; Escada et al., 2009). The olfactory neuronal cells are situated in the olfactory cleft, encompassing the anterior insertion of the middle turbinate, the medial part of the superior turbinate, the cribriform plate, and the superior portion of the nasal septum (Leopold et al., 2000; Pinna Fde et al., 2013). The olfactory mucosa forms part of the olfactory circuit (Borgmann-Winter et al., 2015). This circuit is directly connected to regions of the limbic system, such as the hippocampus and the amygdala, which have been implicated in the pathophysiology of psychiatric disorders (Akil et al., 2018). Based on extensive otolaryngologic research, the optimal site and acquisition techniques for olfactory biopsy have been defined (Feron et al., 2013; Holbrook et al., 2016). In addition, a swab technique has been developed to reduce the invasiveness of the procedure with similar yield of olfactory neuronal cells (Benitez-King et al., 2011). Both techniques allow for ex vivo and in vitro studies of the olfactory neuroepithelium and could be combined with laser-capture microdissection and fluorescence activated cell sorting to increase the yield of olfactory neurons (Narayan et al., 2014).

Depending on the objectives, olfactory tissue can be kept in monolayer cultures or propagated to form primary neurospheres using well established protocols (Matigian et al., 2010). These olfactory neurospheres are multipotent, with the potential for self-renewal and for differentiation into neurons or glia cells (Lavoie et al., 2017). While adult olfactory neurons could be used to study protein abundance, gene expression, or epigenetic signatures, neurosphere cultures could be used to investigate pharmacological responsiveness and cellular function.

#### Olfactory Neurons and Stem Cells as Models for Disorders of the Brain

**—**Over the last decades, the olfactory mucosa has gained increasing attention due to its translational potential and its implications for disorders of the brain (see Table 3) (Borgmann-Winter et al., 2015; Lavoie et al., 2017). This is also reflected by increasing numbers of patents for applications using the olfactory neuroepithelium (Soto-Vázquez et al., 2014) in a variety of these disorders.

**Table 3.** Selection of Findings From Olfactory Neuro-Epithelium–Derived Models for Psychiatric Disorders

Disorder	Model	Results	Reference
Neurodegenerative disorders			
Alzheimer’s disease	Olfactory neuro-epithelial tissue	Methoxy-X04-derivative BSC4090 as a biomarker of early stage Alzheimer	(Pellkofer et al., 2019)
Parkinson’s disease	Cultured olfactory neurosphere-derived cell lines	Disease-specific alterations in gene and protein expression as a model for Parkinson’s disease	(Matigian et al., 2010) (Parkinson + SCZ)
Schizophrenia			
	Cultured olfactory neurosphere-derived cell lines (and iPSCs)	Differences in DNA methylation in fibroblasts, olfactory neurosphere-derived cells, and iPSCs	(Vitale et al., 2017)
	Cultured olfactory neurosphere-derived cell lines	Dysregulated protein synthesis	(English et al., 2015)
	Cultured olfactory neurosphere-derived cell lines	Alterations in cell cycle dynamics	(Fan et al., 2012)
	Olfactory neuro-epithelial tissue	Involvement of the SMAD pathway in brain function	(Horiuchi et al., 2016)
	Olfactory neuro-epithelial tissue	Alterations in cell cycle dynamics	(McCurdy et al., 2006) (SCZ + BP)
	Cultured olfactory neurosphere-derived cell lines	Alterations of microtubular organisation	(Solis-Chagoyan et al., 2013) (SCZ + BP)
	Cultured olfactory neurosphere-derived cell lines	Alterations of autophagic processes	(Sumitomo et al., 2018) (SCZ + BP)
	Cultured olfactory neurosphere-derived cell lines	Changes in IRS2 tyrosine phosphorylation suggestive of insulin resistance	(Takayanagi et al., 2020) (SCZ + BP)
Affective disorders			
Bipolar disorder	Olfactory neuro-epithelial tissue	Alterations in intracellular calcium signaling	(Hahn et al., 2005)
	Olfactory neuro-epithelial tissue	Molecular changes through lithium treatment and association with treatment response	(Narayan et al., 2014)
	Olfactory neuro-epithelial tissue	Association of GSK3ß and CRMP1 with mood symptoms	(McLean et al., 2018)
	Cultured olfactory neurosphere-derived cell lines	Alterations of microtubular organization	(Solis-Chagoyan et al., 2013) (SCZ + BP)
	Olfactory neuro-epithelial tissue	Alterations in phosphatidylinositol signaling pathways	(McCurdy et al., 2006) (SCZ + BP)
Neurodevelopmental disorders			
Fragile-X-Syndrome	Cultured olfactory neurosphere-derived cell lines	Indication for the feasibility of testing for FMR1 mutations using olfactory neurons as a model for Fragile-X syndrome	(Abrams et al., 1999)
Rett-Syndrome	Olfactory neuro-epithelial tissue	Alterations in neuronal structure and quantity as a model for Rett-Syndrome	(Ronnett et al., 2003)
ASD	Olfactory stem cells	Altered molybdenum cofactor sulfenase expression as a potential biomarker for ASD	(Feron et al., 2016)
	Olfactory stem cells	Dysregulation of 4 micro RNAs as early biomarkers of ASD	(Nguyen et al., 2016)
	Olfactory stem cells (and iPSCs)	Impaired expression of a long noncoding RNA (COSMOC) as a model for ASD	(Rontani et al., 2020)

Abbreviations: BP, bipolar disorder; COSMOC, antisense long noncoding RNA of molybdenum cofactor sulfurase (MOCOS); CRMP1, Collapsin Response Mediator Protein 1; FMR1, fragile X mental retardation 1; GSK3ß, Glykogensynthase-Kinase 3; IRS2, insuline receptor subtype 2; SMAD, SMA and Mothers Against Decapentaplegic (MAD); SCZ, schizophrenia.

##### Neurodegenerative Disorders

Alzheimer’s and Parkinson’s disease have been studied using olfactory neuroepithelium (Matigian et al., 2010). For example, Alzheimer pathology, that is, Amyloid-ß aggregate and paired helical filament tau in neurites, is present in the olfactory epithelium, reflecting cortical lesions (Arnold et al., 2010). Moreover, biopsied olfactory mucosa was recently used to develop a potential biomarker for prodromal stages of Alzheimer’s disease (Pellkofer et al., 2019), underlining the great potential of this tissue type.

##### SCZ Spectrum and Other Primary Psychotic Disorders

Regarding SCZ, alterations in cell cycle (McCurdy et al., 2006), protein synthesis (English et al., 2015), redox signaling associated with glutathione cascade (Kano et al., 2013), insulin and metabolic signaling (Takayanagi et al., 2020), protein quality control and autophagy (Sumitomo et al., 2018), and neuronal development (Arnold et al., 2001) and function (Solis-Chagoyan et al., 2013) have been shown. Also, differential gene expression was connected to cognitive functioning using olfactory tissue (Horiuchi et al., 2016). Further, distinct differences in DNA methylation in fibroblasts, olfactory neurosphere-derived cells, and iPSCs from a population of patients suffering from SCZ were found (Vitale et al., 2017). The group did not, however, compare olfactory with blood cells or cerebral tissue.

##### Affective Disorders

So far, 5 groups have used olfactory epithelium-derived tissue of patients with bipolar disorders. Findings include altered expression of a protein kinase through lithium treatment (McLean et al., 2018), differences in intracellular signaling between healthy subjects and medication-free euthymic bipolar patients (Hahn et al., 2005), and shorter microtubules in cultured neurosphere precursors obtained from stable, medicated patients with bipolar disorder type I (Solis-Chagoyan et al., 2013).

##### Neurodevelopmental Disorders

In vitro cultured olfactory neurons were produced to study Fragile X (Abrams et al., 1999) and Rett syndrome (Ronnett et al., 2003). In light of the early disease onset, olfactory stem cells have been used to study ASD, implicating the molybdenum cofactor sulfenase (Feron et al., 2016) and an antisense long noncoding RNA, COSMOC (Rontani et al., 2020), in the etiology of the disorder and 4 miRNAs as potential early biomarkers for ASD (Nguyen et al., 2016).

Even though the threshold to acquire olfactory neuroepithelium in psychiatric patients remains high, these studies highlight the feasibility of obtaining olfactory tissue with no serious adverse events reported.

#### Limitations

**—**Compared with iPSCs and iNs, analysis of olfactory tissue stands out through its relatively low costs, the few necessary modifications needed, and the straight-forward, minimally invasive acquisition to obtain the neuronal tissue. The heterogeneity of the olfactory epithelium can be controlled for using laser capture microdissection or fluorescence activated cell sorting using antibodies against specific markers, such as NeuN (Mullen et al., 1992). Current limitations include the different cellular age of the acquired neurons and the differences in culture and maintenance protocols that could diminish the comparability of the results. Also, one must bear in mind that, while also reacting and responding to stimuli of other transmitter systems, each olfactory neuron is characterized by the expression of 1 specific olfactory receptor (Silva Teixeira et al., 2015). Despite its great potential for translational research, the information gained from studies using the olfactory neuroepithelium should thus be considered “olfactory-specific” and be treated with caution and compared with results from other neuronal models, including iPSCs, cerebral organoids, or post-mortem brains.

### Cerebral Organoids

#### Development of the Model

**—**The term cerebral organoids was introduced by Lancaster et al. in 2013 (Lancaster et al., 2013). The technique was based on the self-organizing potential of human embryonal stem cells and iPSCs to form an interdependent tissue in 3-dimensional Matrigel culture conditions using serum-free growth media with the possibility to recapitulate aspects of human cortical development. Since 2013, the field has seen the optimization of techniques to create region-specific cerebral organoids (Kelava and Lancaster, 2016), such as forebrain tissues, pure midbrain, or hypothalamic tissue, the development of mini-bioreactors to increase production of cerebral organoids as well as the separation of neurons into deep and superficial layers. Also, the organoid-to-organoid comparability and reproducibility has been greatly improved over the last years (Velasco et al., 2019).

#### Cerebral Organoids in Psychiatry

**—**Currently, the focus of research using cerebral organoids is on studying neurodevelopmental disorders and disorders with high heritability estimates (see Table 4).

**Table 4.** Selection of Findings From Cerebral Organoid-Derived Models for Psychiatric Disorders

Disorder	Model	Results	Reference
Neurodegenerative disorders			
Trisomy 21	Cerebral organoids derived from keratinocytes in combination with CRISPR/Cas9	Implication of BACE2 in Trisomy 21 pathology	(Alić et al., 2020)
	Cerebral organoids with PITRM1-knockdown	Association of mutations in metalloproteinase and neurodegenerative proteinopathies	(Pérez et al., 2020)
	Midbrain organoids containing dopaminergic neurons	Observation of disease-relevant phenotypes for Parkinson’s disease	(Reiner et al., 2021) (review)
Substance use disorders			
	Cerebral organoids generated from fibroblasts	Neurotoxic effects of ethanol on metabolic, tissue and cellular levels as model for fetal alcohol spectrum disorders	(Arzua et al., 2020)
Schizophrenia			
	Cerebral organoids	Abnormal distribution of proliferating NPC through different zones potentially associated with FGFR1 signaling	(Stachowiak et al., 2017)
	Cerebral organoids generated from fibroblasts	Dysregulation of genes involved in neurodevelopment and synapse biology	(Kathuria et al., 2020b)
Affective disorders			
Bipolar disorder	Cerebral organoids generated from fibroblasts	Implication of NCAN in bipolar disorder; dysregulated gene expression associated with cell adhesion and immune signaling	(Kathuria et al., 2020a)
Neurodevelopmental disorders			
ASD	Cerebral organoids generated from fibroblasts	Association of transcription factor FOXG1 with overproduction of GABA-ergic in ASD-derived organoids	(Mariani et al., 2015)

Abbreviations: ASD, autism spectrum disorder; BACE2, Beta-Secretase 2; FGFR1, Fibroblast growth factor receptor 1; FOXG1, Forkhead box protein G1; GABA, gamma-aminobutyric acid; NCAN, Neurocan; PITRM1, pitrilysin metallopeptidase 1.

##### Neurodegenerative Disorders

Building on insights gained from iPSC research, it is now possible to combine the reprogramming technology with CRISPR-Cas9 and create cerebral organoids with trisomy 21 (Alić et al., 2020) and features of sporadic Alzheimer’s disease. Using mathematical models, drugs can then be tested in the sense of a precision medicine (Park and Jang, 2021). Also, cerebral organoids have been used to investigate the association of mutations in metallopeptidases and neurodegenerative proteinopathies (Pérez et al., 2020). With the development of midbrain organoids, the investigation of Parkinson’s disease becomes increasingly possible, already enabling the observation of disease-relevant phenotypes (Reiner et al., 2021).

##### Substance Use Disorders

As a model for fetal alcohol spectrum disorder, the neurotoxic effects of ethanol exposure on metabolic, tissue, and cellular levels were shown using 2-month old iPSC-derived human cerebral organoids (Arzua et al., 2020).

##### SCZ Spectrum and Other Primary Psychotic Disorders

In light of dysregulated neuronal development, cerebral organoids were used to provide insights into early brain development in SCZ, implicating a loss of nuclear FGFR1 with impaired cortical development (Stachowiak et al., 2017) and a dysregulation of genes involved in neurodevelopment and synapse biology (Kathuria et al., 2020b) as disease phenotypes of SCZ.

##### Affective Disorders

In cerebral organoids from bipolar patients and healthy individuals, dysregulation of genes implicated in immune signaling and impairments in neuronal transmission were reported (Kathuria et al., 2020a).

##### Neurodevelopmental Disorders

Using dorsal telencephalic organoids from patients with ASD and macrocephaly, an upregulation of FOXG1, important for forebrain development regulation, was found compared with healthy controls (Mariani et al., 2015). Furthermore, an increase in GABA-ergic neurons in the ASD organoids was shown. Cerebral organoids have also been used in combination with CRISPR/Cas9 to investigate the influence of candidate genes on gene expression and cortical neurogenesis (Wang et al., 2017a; Zhang et al., 2020). These genes might represent molecular targets for drug development.

#### Limitations

**—**Despite the great advances over the last years, including improved comparability, reproducibility (Lancaster et al., 2017), and vascularization (Pham et al., 2018), the generation of cerebral organoids remains a process requiring a specialized laboratory with optimal culture conditions and an adequate standard of technical expertise to ensure low heterogeneity and variability between organoids. Cerebral organoids remain smaller than the human brain due to the insufficient supply with oxygen and nutrients in culture conditions (Liu et al., 2019). In addition, the process continues to be time consuming and expensive (Parr et al., 2017). When developing new models for brain network disorders, these limitations should be kept in mind and clear hypotheses generated to save resources and increase cost-effectiveness.

## Implications and Future Perspectives

As experimental models, iPSCs, induced neurons, cerebral organoids, and olfactory neuronal cells have greatly advanced psychiatric research over the last decade.

iPSC-derived neuronal subtypes already enable the high-throughput screening for genetic and pharmacological targets (Amin and Pasca, 2018). In addition, iPSC-derived 3-dimensional cerebral organoids allow for the study of cortical development and network formation encompassing different neuronal subtypes. The process of transdifferentiation also produces neuronal precursors and subtypes, with the advantage of a retained epigenetic memory. The same holds true for olfactory neurons and stem cells, neuronal subtypes that are easily accessible and that can be obtained repeatedly and minimal invasively.

Advances in the development of viable models and the diagnostic possibilities will go hand in hand, most preferably by combining the abovementioned cellular models. At initial manifestation of symptoms, iPSCs and iNs could be generated and the methylome of olfactory neurons obtained. Similar to antibiotic algorithms (Morales et al., 2018), treatment might be started “empirically” as soon as all necessary information has been obtained. In the meantime, the cell cultures would be tested for their reactiveness to different classes of drugs. This reactiveness could be correlated with the specific symptoms, established psychometric scales, or the recently defined research domain criteria (Cuthbert, 2014) to generate a patient-specific model (termed, e.g., depressiogram). Based on this model, the treatment strategy might be adopted accordingly to reflect the neuronal signatures. Combined with psychometric scales, epigenetic patterns of olfactory neurons could be used as parameters of treatment response. The responsiveness of iPSC-derived neuronal subtypes and cerebral organoids to medication could also be correlated with neuroimaging techniques, including positron emission tomography.

Before these models can be implemented to reliably study greater patient cohorts, some challenges remain, such as the implementation of methodologic standards and the improvement of comparability and reproducibility of the techniques (Brennand et al., 2015). This will also include comparing the behavior of iPSCs, iNs, and olfactory neurons in different culture media and conditions and investigating the non-diseased reaction of these neurons to medication as well as comparing patients suffering from different disorders (e.g., patients suffering from MDD and bipolar depression).

The abovementioned cell types and techniques hold great promise to increase the accuracy of current experimental models. This will lead to a further approximation to the tissue of interest and to the implementation of viable explanatory models for psychiatric disorders, thereby also eventually reducing the substantial burden of disease currently caused by psychiatric disorders (Rehm and Shield, 2019).

## Statement of Interest

Without any relevance to this work, R. Lanzenberger received travel grants and/or conference speaker honoraria within the last 3 years from Bruker BioSpin MR and Heel and has served as a consultant for Ono Pharmaceutical. He received investigator-initiated research funding from Siemens Healthcare regarding clinical research using PET/MR. He is a shareholder of the start-up company BM Health GmbH since 2019. J. Unterholzner, V. Millischer, C. Wotawa and A. Sawa declare no conflicts of interest.
